# Toothache

**Published:** 1898-11

**Authors:** 


### Toothache.

Dr. Smith considers Phenacetin as the safest of all remedies for relieving odontalgia. He usually prescribes five-grain doses, with a third of a grain of digitalis, and gets very good results. Five grains every three hours until three doses have been taken, and not to renew the dose until several hours afterwards.—*Items of Interest*, June, 1898.

